# Notch Corrosion Fatigue Behavior of Ti-6Al-4V

**DOI:** 10.3390/ma7064349

**Published:** 2014-06-11

**Authors:** Sergio Baragetti

**Affiliations:** 1 GITT - Centre on Innovation Management and Technology Transfer, University of Bergamo, Via Salvecchio 19, Bergamo 24129, Italy; E-Mail: sergio.baragetti@unibg.it; 2 Department of Engineering, University of Bergamo, Viale Marconi 5, Dalmine 24044, Italy

**Keywords:** Ti-6Al-4V, corrosion fatigue, high notch, *K_t_* effect

## Abstract

The aim of this paper is to map the corrosion fatigue characteristics of Ti-6Al-4V alloy through the evaluation of the corrosion fatigue initiation and failure mechanisms. The study included the effect of the stress concentration factor at very high *K*_t_ values and the role of different inert or corrosive environments. This alloy is widely used in naval-structures and aero-engine communities and the outcomes of the work will have direct relevance to industrial service operations. Axial fatigue tests (*R* = 0.1; 2 × 10^5^ cycles; *f* = 10 Hz) were carried out on smooth and high notched (*K*_tmax_ = 18.65) flat specimens in laboratory air, paraffin oil, laboratory air + beeswax coating, recirculated 3.5% NaCl solution. The step loading procedure was used to perform the fatigue tests and the surface replica method and crack propagation gages were used to check crack nucleation and propagation until failure. Log-Log plots of σ_max_ *vs.* *K*_t_ showed a bilinear behavior and enabled the demonstration of the presence of a threshold stress intensity factor (*K*_t_ = 8–9), after which the environment has no effect on the fatigue damage for all the tested environments.

## 1. Introduction

High strength/mass ratio mechanical or aeronautical components that work in an aggressive environment need the employment of light alloys or composite materials. Combined with their low density and intermediate to high strength, Titanium alloys offer clear advantages for naval-structures, aero and marine power, off-shore, biomedical and chemical processing applications [1].

Data for smooth and notched (with low stress concentration factors) Ti-6Al-4V samples, fatigue tested in laboratory air or aggressive environment, are available in the literature [2,3,4,5,6,7,8,9,10,11], while only in a few references can the results at high stress concentration factors and in a very aggressive environment be found [12,13,14,15,16,17,18].

In [2], Morrissey and Nicholas tested cylindrical dogbone samples under fully reversed loading (*R* = −1) with an upper limit of 10^9^ load cycles. The material mechanical properties were: *UTS* = 968 MPa and *YS* = 930 MPa. The tests were carried out at 60 and 20 kHz. After around 200,000 load cycles, the fatigue limit of the Titanium alloy is approximately constant regardless of the test frequency. In [3], Bellows *et al.* used a step loading procedure to carry out axial fatigue tests at 10^7^ cycles on smooth cylindrical Ti-6Al-4V samples. The specimens were formed from fan blade forgings with their axis parallel to the longest direction in the forging. The material *UTS* and *YS* were 978 and 930 MPa, respectively. *R*-ratios equal to −1, 0.1, 0.5 and 0.8 were investigated and the test frequency was 60 Hz. Room temperature endurance limits and constant-life Haigh (modified-Goodman) diagrams for smooth Ti-6Al-4V specimens generated by both the step method and conventional method (using *S-N* curves) were analyzed and statistically compared. The conclusion is that step testing yields results that are within the statistical limits of conventional S-N curve results and therefore is a valid method for generating endurance limits and therefore Haigh diagrams for Ti-6Al-4V specimens.

In [4], D.B. Lanning *et al.* tested cylindrical samples machined from forged Ti-6Al-4V plate along the longitudinal direction. The mechanical properties in this direction were: *UTS* = 978 MPa and *YS* = 930 MPa. Both smooth and V-notched (*K*_t_ = 2.0, 2.7 and 4.1) cylindrical samples were tested under axial fatigue with *R*-ratios = −1, 0.1, 0.5, 0.65 and 0.8. The samples were all stress relieved after the machining operations. The fatigue limit at 10^6^ cycles was estimated using the step loading method [3] at a test frequency of 50 Hz. Finite element solutions were generated to provide stress distributions for the notched gage sections. The stress distributions were used in the search for a critical distance over which the quantities of mean stress, stress range, or elastic strain energy may contribute to the fatigue process and can be correlated to similar quantities from smooth, unnotched specimens.

In [5], D.B. Lanning *et al.* tested notched cylindrical samples machined from a Ti-6Al-4V forged plate in STOA condition under axial fatigue (40 Hz) at 10^6^ cycles. The material mechanical properties in the longitudinal direction were: *UTS* = 978 MPa and *YS* = 930 MPa. The samples were all stress relieved after the machining operations. Stress concentration factors of 1.97, 2.72, 2.85, 2.86, 4.07 were obtained and *R*-ratios = −1, 0.1, 0.5, 0.65 and 0.8 were investigated. A step loading procedure with a stress level increment of 5% of the previous step was implemented for generating points on a Haigh diagram. The experimental data were used in combination with finite element solutions for all specimen geometries to determine a “critical distance” parameter, determined from the stress distribution surrounding the notch in combination with fatigue limit stress data from unnotched specimens, that allows fatigue resistance evaluation.

**Figure 1:**
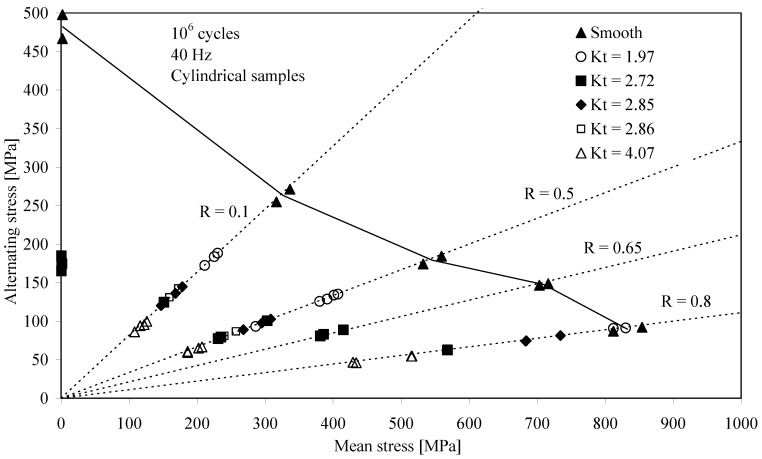
Haigh diagram for a constant life of 10^6^ cycles; smooth and notched Ti-6Al-4V samples [4,5].

Figure 1 shows the collected results [4,5].

**Figure 2:**
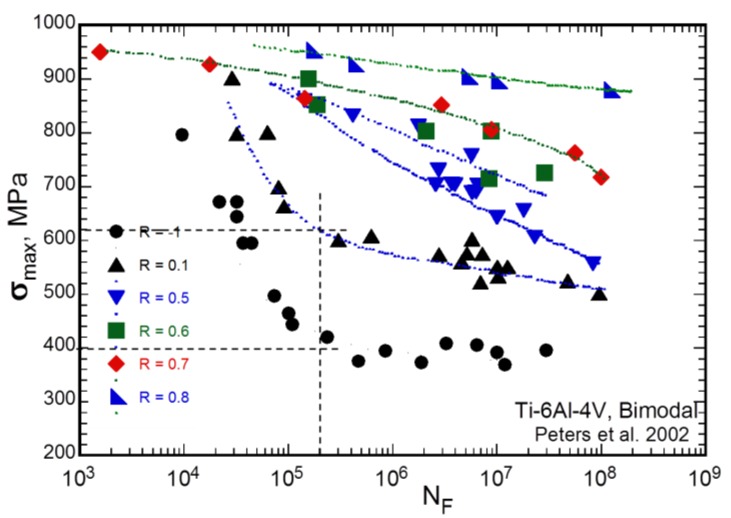
*S-N* data for Ti-6Al-4V alloy plotted in terms of σ_max_ *vs.* the number of cycles to fail for various constant *R*-ratios [11].

In [11], experimental data on fatigue tests on smooth cylindrical samples are reported. The tests were carried out on smooth hourglass samples machined from a bar. The Titanium alloy considered was a bimodal Ti-6Al-4V alloy with *UTS* = 965 MPa and *YS* = 915 MPa. The test results are collected in the diagram of Figure 2, where the limiting stress at 2 × 10^5^ cycles for *R* = −1 and 0.1, respectively, are also indicated. The data collected for *R*= −1 are in good agreement with the literature references mentioned in previous reports [2]. The results also show that after around 100,000 load cycles the fatigue limit of the Titanium alloy is approximately constant regardless of the number of cycles. In Bellows *et al.* [3], the fatigue limits were also approximately constant with the number of cycles for a loading ratio *R* = 0.1.

The effect of a very aggressive environment, such as a methanol solution, has not been accurately tested yet, notwithstanding its relatively great importance in practical applications. Johnston *et al.* [15] made an analysis of the failures occurred in pressurized aerospace methanol tanks. Test results indicated that the alloy is highly notch-sensitive in methanol. Stress-corrosion cracking in the alloy when exposed to methanol was also observed.

Although Titanium is a highly reactive material, Titanium alloys typically exhibit a high resistance to corrosive environments, due to the presence of a layer of Titanium oxide (TiO_2_) which tends to passivate the action of the external environment [16]. However, the influence of a mechanical action or abrasive can remove this surface layer, generating a direct interaction between the Titanium alloy and the external environment, leading to the appearance of relevant phenomena of stress corrosion cracking in an aqueous environment [17]. This interaction is also generated by the presence of surface discontinuities, such as cracks, damage and notches, which break the continuity of the passivating layer of oxide, generating in this case also the susceptibility to stress corrosion cracking in water [13,14].

This trend was also found in environments other than water, highlighting in particular the high sensitivity of Titanium alloys to stress corrosion cracking in pure methanol if there are efforts made and there is the presence of cracks or notches, as observed in the failure of tanks of methanol used in the aerospace field [15]. The addition of water in solution with methanol, however, seems to inhibit these effects, allowing the new formation of oxides that protect the surface against the corrosive effect of methanol on this and other Titanium alloys, as has been observed on the alloy Ti-6Al-4V [16,17].

Recent experiments on the fatigue behavior of slightly notched (*K*_t_ = 1.18) Ti-6Al-4V specimens immersed in solution at various concentrations of methanol, however, have shown that, in the presence of dynamic loads applied (*R* = 0.1), the effect of corrosion appears to be significant, even for very high amounts of water in solution [18]. There is also an obvious correlation between the concentration of methanol and the breaking stress. This marked sensitivity of Titanium alloys exposed to mixtures of water and methanol can give rise to safety problems in the aeronautical field, since the injection of such mixtures in the compression stage of the turbine engine is used to retrieve the performance under conditions low density outside air [19]. These problems can be extended to other structural elements made of Titanium alloy, whereas the majority of aircraft components is constantly fatigue loaded, for the heavy and repeated dynamic loads to which aircraft are typically subjected. The few experimental results related to the study of Titanium alloys in a solution of water, NaCl solution and methanol environment, presented in [10,15,16,18], and related to different geometries of the specimens, do not allow to quantify, at the project level, the actual margin of safety for the design of components in Ti-6Al-4V.

The aim of this paper is to evaluate the environmentally assisted crack growth in a single Titanium alloy, Ti-6Al-4V alloy widely used in naval-structures and aero-engine communities, whilst gaining a fundamental understanding of corrosion mechanisms that should be applicable to other Titanium alloys of this class. In this respect, the outcomes of the project will have direct relevance to industrial service operations. The study included the effect of the stress concentration factor for very high values of *K*_t_ and the role of inert or corrosive environment. The Titanium alloy was tested in several environmental conditions in order to study the fatigue crack initiation/growth mechanisms under: (1) recirculated 3.5% NaCl solution; (2) inert environments like laboratory air, paraffin oil, laboratory air + beeswax coating. The samples were machined with a sharp notch having a stress concentration factor *K*_t_ ranging from 1.16 to 18.65. Such tests would allow to decouple the environmental and stress effects and understand the corrosion fatigue mechanism in terms of chemical and mechanical driving forces. More than 50 flat smooth and V-notched specimens (*K*_t_ = 1.16–18.65) made of Ti-6Al-4V (ASTM B265-99 [20]; STOA treatment; *UTS* = 990 MPa; *YS* = 945 MPa) were tested. The test time for each sample was about 1 week for a total testing time of 70 weeks (very long time experiments).

## 2. Experimental Techniques

### 2.1. Material and Samples Geometry

Axial fatigue tests (*R* = 0.1), 2 × 10^5^ cycles, *f* = 10 Hz were carried out on Ti-6Al-4V smooth and notched flat specimens. The fatigue tests were carried out on mild notched and notched specimens in different inert or aggressive test environments: laboratory air (relative humidity 30%; *T* = 22 °C), paraffin oil, laboratory air + beeswax coating and recirculated 3.5% NaCl solution.

**Figure 3:**
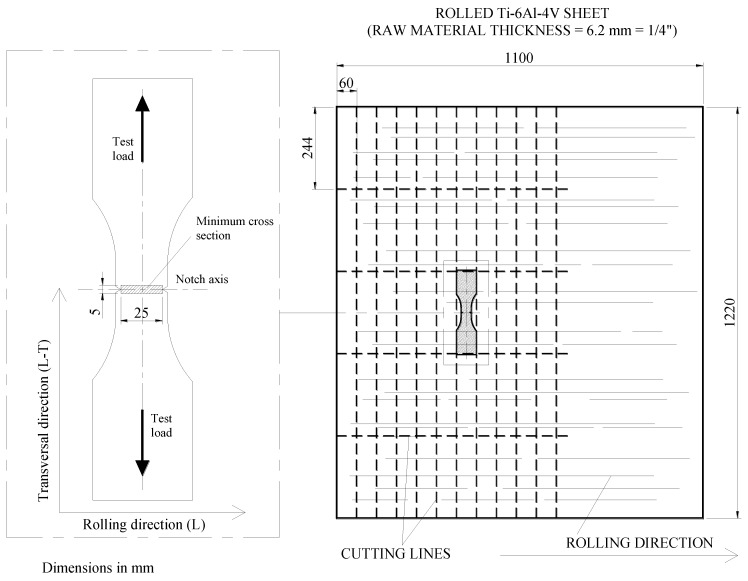
Sketch of the specimens’ orientation with respect to the parent Ti-6Al-4V plate rolling direction.

The samples were prepared so that the loading axis was transversal to the rolling direction of the parent Ti-6Al-4V plate. Figure 3 shows the sketch of the V-notched samples and orientation with respect to the parent Ti-6Al-4V plate rolling direction. The samples, after the forming operations, were STOA heat treated. The STOA treatment consisted of solution treatment at 925 °C (1 h) and vacuum annealing at 700 °C (2 h) for stabilization.

**Figure 4:**
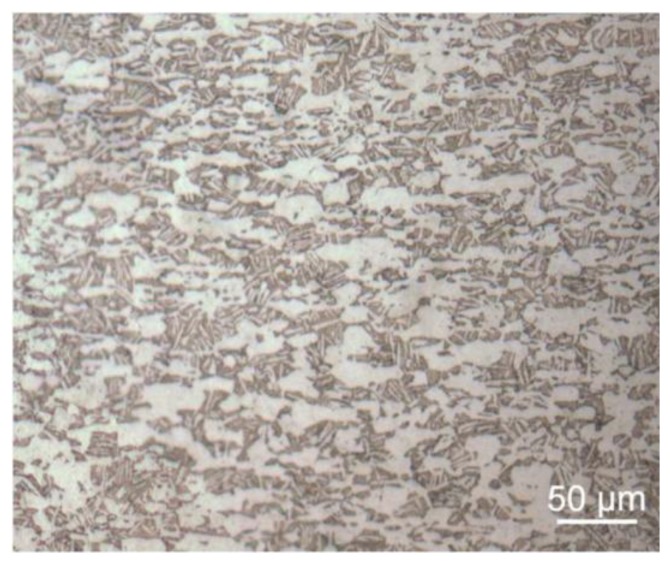
Bimodal microstructure of Ti-6Al-4V after the STOA treatment. Magnification: 200×.

The samples were machined from two plates made of Ti-6Al-4V (ASTM B265-99 [20]; STOA treatment; *UTS* = 990 MPa; *YS* = 945 MPa) and then were subjected to STOA treatment. This treatment consisted in a solution treatment at 925 °C (1 h) and vacuum annealing at 700 °C (2 h) for stabilization. Figure 4 shows the bimodal α + β microstructure of a piece of sample. The average primary alpha grain size is about 30 µm. By image analysis the amount of primary alpha grains was estimated equal to about 50%.

Figure 3 shows the geometry of the V-notched samples (*K*_t_ = 1.16–18.65). The values of the numerical *K*_t_ for each notch tip radius ρ is reported in Table 1. The notches were obtained by milling at very low cutting speed to limit the residual stresses, then by electrical discharge machining (EDM) to obtain the precise values of the different notch root radii ρ. All the samples were finally stress relieved at 700 °C in a vacuum for 1 h.

**Table 1:** Values of the numerical *K_t_* for each notch tip radius ρ.

Types	Values of the numerical *K*_t_ for each notch tip radius ρ								
ρ (mm)	30	30	2.5	1.5	0.45	0.26	0.06	0.05	0.025
*K*_t_	1.16	1.18	2.55	3.10	5.17	6.63	13.34	14.34	18.65

### 2.2. Experimental Equipment and Procedures

The step loading procedure [3,4] was used for the axial fatigue tests on flat smooth and notched Ti-6Al-4V samples both in laboratory air and in aggressive or inert environments.

The main features of the test procedure are the listed below:

(1) A reference step number of cycles equal to 200,000 was considered;
(2) Only two specimens were needed for each experimental condition. The second specimen was the confirmation one and was tested at the fatigue limit found with the first step loaded specimen;
(3) Reference Haigh diagrams extrapolated from the literature were used to define the initial test loads. Each initial test load was defined so that the applied maximum stress was calculated by lowering by a certain percentage the limiting stress condition from the diagram;
(4) The loading condition of each block was defined by increasing by 5%–10% the maximum load of the previous one;
(5) Every 10,000–20,000 load cycles of each loading block replicas were taken at the notch tips on both the sample faces until the nucleation occurrence has been noticed. After the nucleation the replicas were taken more frequently. The procedure was carried on until the complete failure of the sample;
(6) The fatigue limit was calculated by interpolating between the last two stress levels according to equation (1) [3,4]:

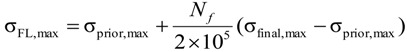
(1)

The duration of the steps chosen for the step loading procedure corresponds to the number of cycles at which the fatigue limit is calculated. Therefore, by considering that the universal testing machine available for the tests can work at a maximum frequency of 15 Hz, steps of the order of 200,000–500,000 cycles—*i.e.* included in the previously mentioned range—need about 4–9 h to be completed. As a result, if a fatigue limit at a number of cycles between 200,000 and 500,000 cycles were selected for the tests, considerable time-saving would be allowed.

**Figure 5:**
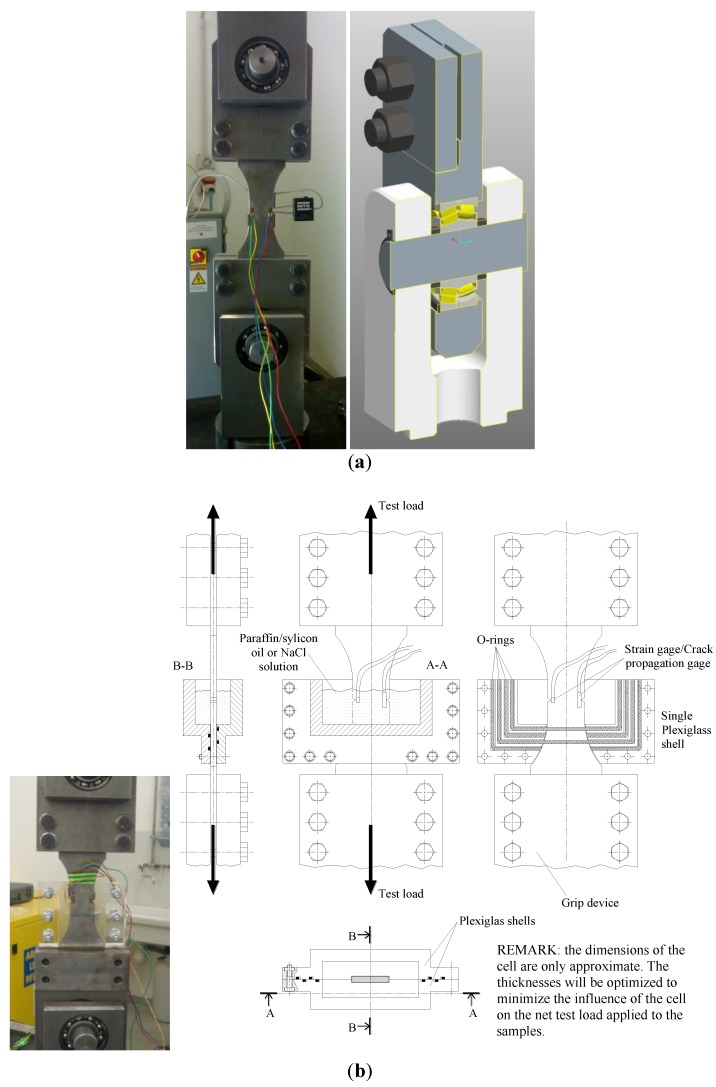
(**a**) Picture of a specimen mounted on the gripping devices; and (**b**) detail of the closed cell mounted on a specimen during the tests.

Gripping devices were developed in order to avoid any parasite bending moment on the specimens (Figure 5a). The maximum allowable force is 150 kN. Suitable constraining systems (bearings) were included in these assemblies to prevent the sample from parasite bending stresses while being fixed in the machine.

A watertight cell with a suitable recirculation system was assembled for the tests in NaCl solution so that the sample notch area was completely submerged in the corrosive medium, which was continuously pumped through the recirculation circuit. Figure 5b shows the sketch of the system and a detail of the cell, respectively. The system was composed of a closed cell equipped with two corrosion-resistant threaded rapid tubing connections, a solution reservoir, a pump and suitable piping connections. The cell consisted of two Plexiglass shells joined by bolting and sealed with sealing filler. The shells could be easily separated and joined together again whenever surface replicas had to be taken during the fatigue tests. In order to prevent an increase of the internal pressure during the tests, the diameter of the outlet pipe was greater than the diameter of the inlet one.

**Figure 6:**
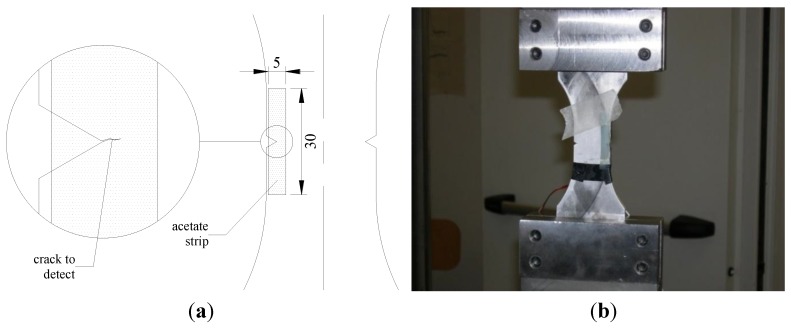
(**a**) Sketch of an acetate strip for surface replication placed over the notch root area; and (**b**) picture of the acetate strip on the specimen.

The features at the notch tip were also controlled during the tests by means of the replica method and by using crack propagation gages. This technique was successfully applied to observe when the fatigue crack initiation occurred and to assess the growth rate of the propagating crack. Replicas were taken for both the notches of the samples every 10,000 or 20,000 load cycles. Replica were taken after nucleation until complete fracture. After nucleation the interval was set at 5,000 load cycles. The replica material was acetate in sheets having a thickness of the order of a few tenths of mm. Small strips (5 mm × 30 mm) of acetate were cut to cover the area of interest in the way shown in Figure 6.

**Figure 7:**
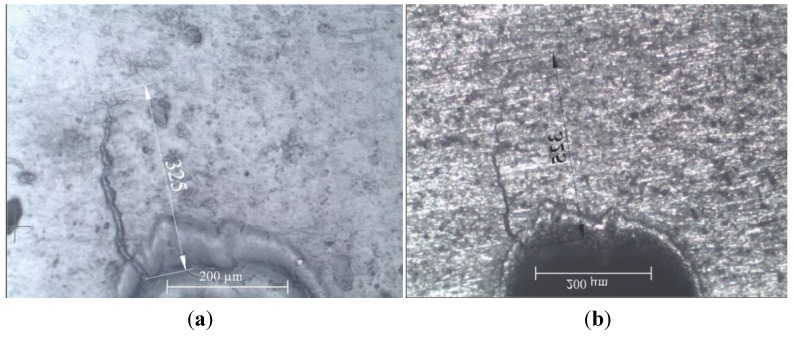
(**a**) Surface replica; and (**b**) microscope observation of the sample in the same area (mirror image).

Before placing the acetate strip over the notch root area it was softened with acetone by immersion for about 5 s. Both the dimensions of the strips and the duration of the immersion were calibrated to prevent the risk of too large deformations and bulginess of the replicas after the complete evaporation of the solvent. After immersion, the strips were firmly pressed into position on the polished metal surface. An accurate preparation of the surface to investigate was necessary to produce replicas able to show all the features accurately. The replicas remained on the notch tip area for at least 20 s in order to let the soft acetate penetrate into the voids present over the surface, so that the image of possible cracks could be captured. Then the replicas were carefully removed from the metal surface and left in a safe place until the solvent evaporation had been completed. Each replica was labelled and addressed to optical microscope observation (Leica DFC290). At least two or three replicas have to be produced for each notch and sample face to compare the results with one another and hence to maximize their reliability. This reliability can conveniently be improved further on with periodic comparison of the replicated crack depths with micrographs of the sample over the crack area. Examples of the surface replica detection are shown in Figure 7.

**Figure 8:**
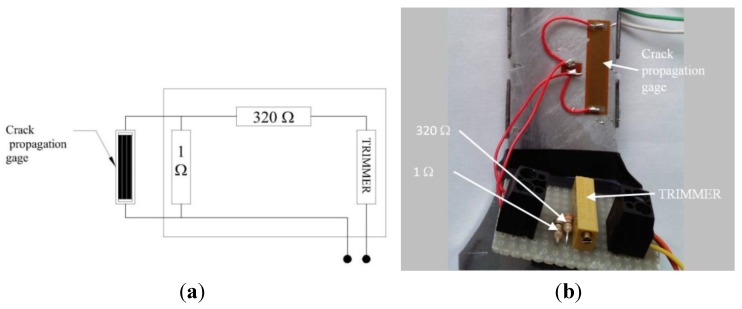
(**a**) Sketch of the electrical circuit including the CPG; and (**b**) picture showing the circuit assembled with the indication of each component.

Crack propagation gages (CPG-HBM RDS22) were attached at a notch root area of the samples to evaluate the opportunity of recording the crack growth rate while the tests were running. These gage patterns consist of a number of resistor strands connected in parallel and their orientation must be transversal to the crack propagation direction. The propagation of the surface crack through the gage strands causes a CPG progressive open-circuiting which results in a progressive increase of the total resistance. The chosen crack propagation gage had an initial resistance of about 0.88 Ω. As a result, each strand fracture was expected to cause an increment of the resistance equal to 0.018 Ω. In order to enable the amplifier to acquire the signal correctly, the circuit including the CPG was arranged in the way shown in Figure 8. A constant resistance of 350 Ω was selected for the second bridge leg and a trimmer operating in the range ±50 Ω was used to equilibrate the Wheatstone half bridge. In fact, this device allowed fine adjustment of the resistance of the leg including the CPG to the value of 350 Ω. No thermal compensation was considered since the tests were conducted in a laboratory conditioned room.

**Figure 9:**
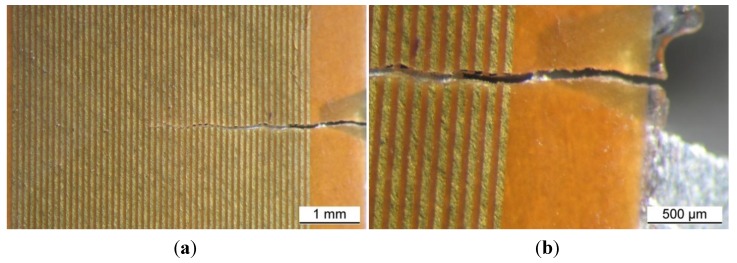
(**a**) Micrograph (20×) of the crack propagation gage (about 30 strands are broken); and (**b**) detail (50×) of the crack propagation gage at the notch root area: the fatigue crack crosses only the first 4 strands of the crack propagation gage. Each strand rupture is related to the increment in crack propagation (with constant distance between the strands).

Examples of the crack detection using crack propagation gages are shown in Figure 9.

## 3. Results and Discussion

**Figure 10:**
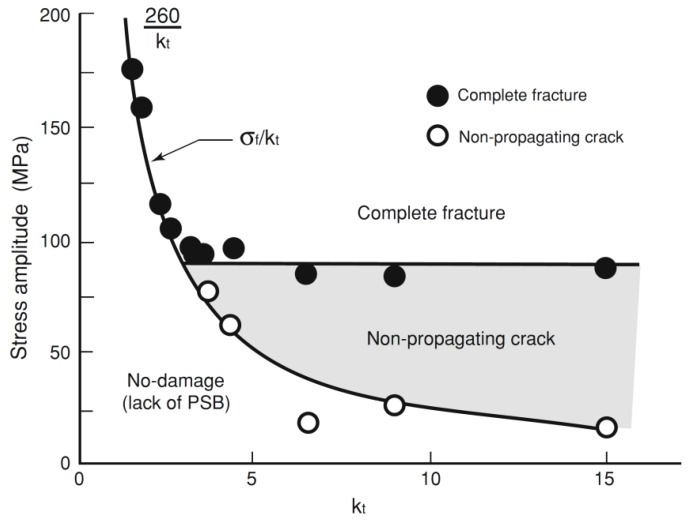
Nominal alternating stress *vs.* *K*_t_ [7,21].

The experimental results were collected and converted into data points on a Frost and Dugdale-like diagram [7,21] obtained for mild steel at *R* = −1 (Figure 10), which shows the fatigue limit alternating stress at nucleation and complete fracture *versus* *K*_t_. With *K*_t_ ranging from 1 to 15, Frost and Dugdale [7,21] found that a critical threshold stress concentration factor, approximately equal to three, is present for mild steel. After such value of *K*_t_ the complete fracture line and the non-propagating cracks line bifurcate, giving evidence of a lower effect of the stress concentration factor on the fatigue resistance after such threshold. The critical stress concentration factors in different environments, given by the intersection between the complete fracture lines and the initiation curves, in this papers were estimated for the Titanium alloy.

**Figure 11:**
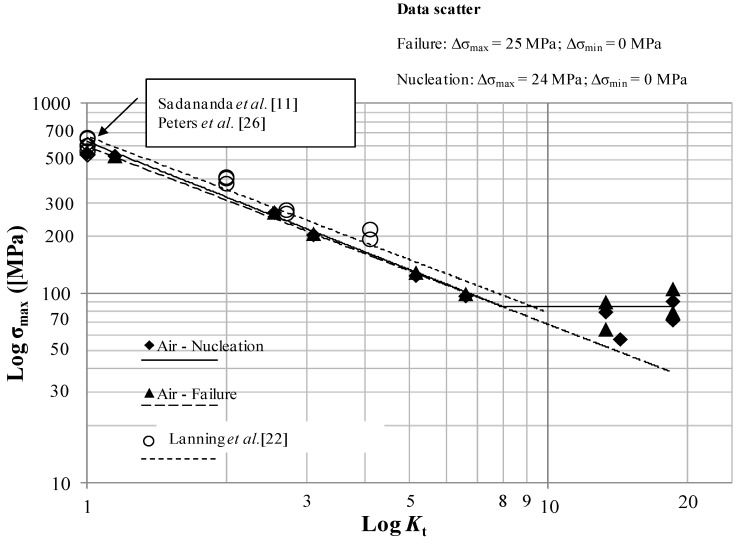
Log-limiting maximum stress (*R* = 0.1, 10 Hz) at initiation and failure *vs.* Log-*K*_t_ at a constant life of 200,000 cycles for the samples tested in air.

**Figure 12:**
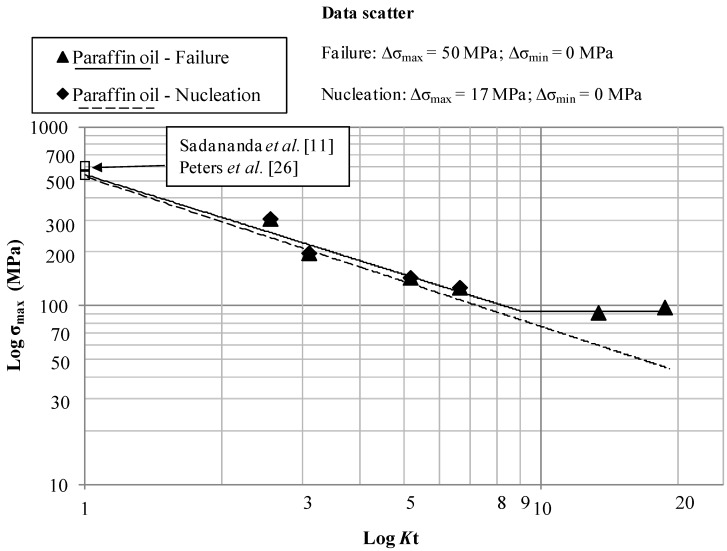
Log-limiting maximum stress (*R* = 0.1, 10 Hz) at initiation and failure *vs.* Log-*K_t_* at a constant life of 200,000 cycles for the samples tested in Paraffin oil.

**Figure 13:**
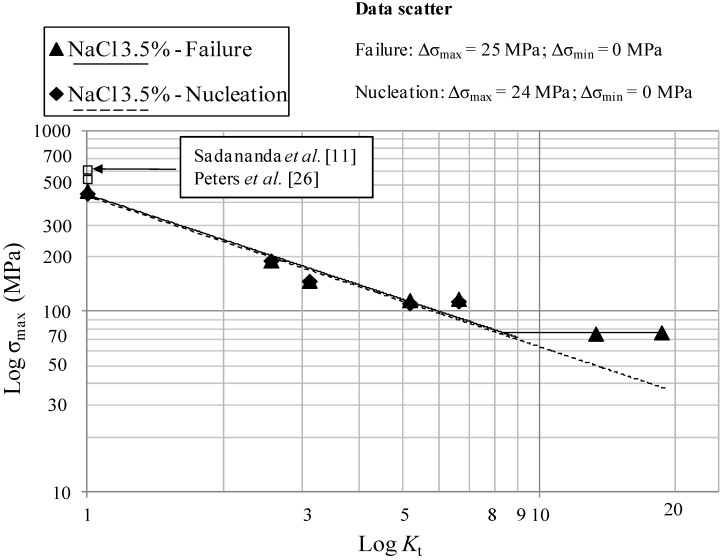
Log-limiting maximum stress (*R* = 0.1, 10 Hz) at initiation and failure *vs*. Log-*K*_t_ at a constant life of 200,000 cycles for the samples tested in NaCl 3.5 wt%.

The main results for the tests in air, paraffin oil, used to simulate an inert environment, and 3.5 wt% NaCl solution, are summarized in Figure 11, Figure 12 and Figure 13 where the Log-limiting maximum stress (*R* = 0.1) at initiation and failure *vs.* Log-*K*_t_ diagram at a constant life of 200,000 cycles for the samples tested in air, Paraffin oil and 3.5% NaCl solution are shown respectively.

The interpolating lines in Figure 11 (tests in air) have the following equations:

σ_max_ = 627*K_t_*^−0.964^ MPa; 1 ≤ *K_t_* ≤ 8   ailure (2)

σ_max_ = 84 MPa; *K_t_* > 8   Failure (3)

σ_max_ = 670*K_t_*^−0.930^ MPa; 1 ≤ *K_t_* ≤8   Failure, Lanning *et al.* [22] (4)

σ_max_ = 592*K_t_*^−0.938^ MPa   Nucleation (5)

The interpolating lines in Figure 12 (tests in Paraffin oil) have the following equations:

σ_max_ = 544*K_t_*^−0.801^ MPa; 1 ≤ *K_t_* ≤ 9   Failure (6)

σ_max_ = 94 MPa; *K_t_* > 9   Failure (7)

σ_max_ = 529*K_t_*^−0.84^ MPa   Nucleation (8)

The interpolating lines in Figure 13 (tests in NaCl 3.5 wt%) have the following equations:

σ_max_ = 443 × *K_t_*^−0.832^ MPa; 1 ≤ *K_t_* ≤ 9   Failure (9)

σ_max_ = 76 MPa; *K_t_* > 9   Failure (10)

σ_max_ = 433 × *K_t_*^−0.833^ MPa; *K_t_* ≤ 9   Nucleation (11)

The limit stresses in air and paraffin oil, shown in Figure 11 and Figure 12, are nearly the same, thus confirming that laboratory air and the inert paraffin oil environment exert the same damaging effect. Beeswax was used too in order to simulate the inert environment but the results were the same as the ones obtained in air and paraffin oil. The percentage of reduction of the fatigue maximum stress in 3.5% NaCl solution is approximately 20% with respect to air and paraffin oil, thus indicating a shift of the curves towards lower stresses in case of tests in 3.5% NaCl solution.

There is a good agreement between the literature values of the limiting maximum stresses at *K*_t_ = 1 (Sadananda *et al.* [11], Peters *et al.* [23]) as shown in Figure 11 and Figure 12.

Log-Log plots show a bilinear behavior in different environments and a threshold *K*_t_ = 8–9 after which σ_max_ remains constant, regardless of *K*_t_. After the threshold *K*_t_ there is a region of non-propagating cracks between crack initiation and crack failure, in accordance with the literature for other materials at lower *K*_t_ [24]. Referring to [7], in which Frost and Dugdale fatigue tested flat mild steel smooth and notched specimens, a relatively low threshold *K*_t_ equal to three was found. This Titanium alloy shows a different behavior both in inert, air and in corrosive environments. This means that, regardless of *K*_t_, if *K*_t_ is higher that 8–9, σ_max_ remains constant for a notched component and the environment does not have a great influence on the fatigue resistance. This seems to be a relative important result in case of the aeronautical Ti-6Al-4V components that work in aggressive environments like a 3.5% NaCl solution. The non-propagating cracks curve and the almost triangular area after bifurcation of the curve after the *K*_t_ = 3, clearly visible in Figure 10 and obtained for a mild steel, is equal to 8–9 in Figure 11, Figure 12 and Figure 13 for Ti-6Al-4V. Furthermore, crack nucleation and complete failure curves are very close for Ti-6Al-4V. The Titanium alloy exerts a much higher crack propagation rate [24] with respect to mild steel, for all the *K*_t_ and test environment considered in the present paper. This means that after nucleation the crack rapidly propagates until complete fracture of the specimens. Moreover, crack growth rates in 3.5% NaCl solution proved to be higher than they were in air at all the *K*_t_ investigated [24].

Due to the high notch sensitivity of the material and to the rather high crack propagation rate, it was quite difficult to more accurately detect crack initiation by using the replica method and crack propagation gages. Nucleation is mainly controlled by the maximum stress at the notch tip: the time for nucleation is the same if the maximum stress at the notch is the same. Fatigue crack propagation until failure depends on the stress gradient and on the applied load. The potential drop technique [25] might prove to be useful to detect with a higher accuracy crack initiation and better describe what happens after the threshold *K*_t_.

**Figure 14:**
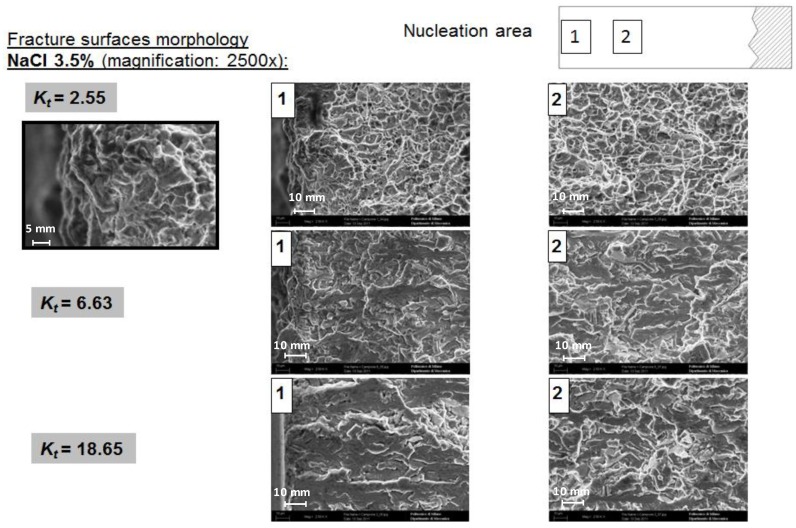
Fracture surface morphology: effect of *K*_t_ [26].

The SEM analyses highlighted different fracture surface features for different *K*_t_ in 3.5 wt% NaCl solution (Figure 14). Between the two points (1 and 2) of the fracture surfaces there is no difference of morphology. As *K*_t_ increases the fracture surface becomes smoother and less corrugated with no dimples. The explanation is that lower *K*_t_ means higher applied loads in order to reach the stress for crack nucleation at the notch tip; on the other hand, because of the steep stress gradient, higher *K*_t_ means lower applied loads in order to reach the stress for crack nucleation at the notch tip.

**Figure 15:**
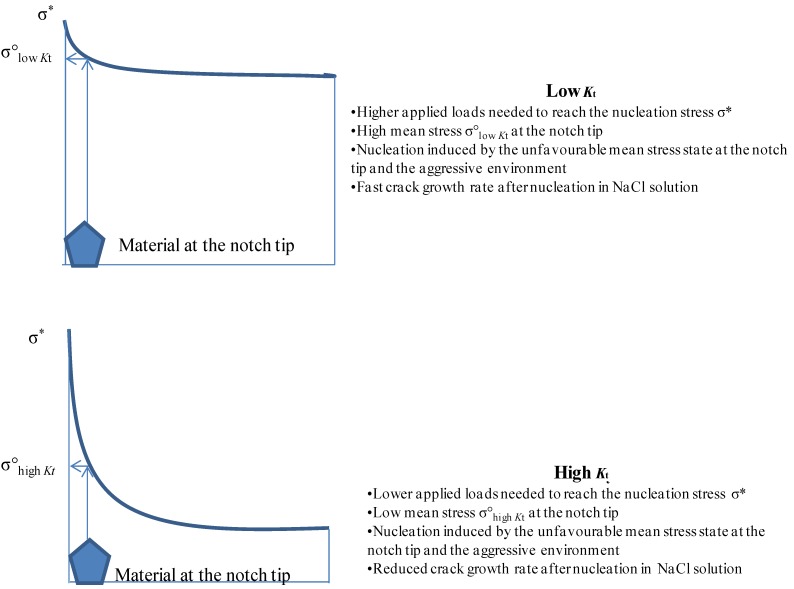
Crack nucleation and propagation: stress gradient and environmental effects.

The observed behavior and the presence of the threshold *K*_t_ is surely strongly dependent on a lot of parameters among which the most important might be cyclic frequency, microstructure and the notch depth. For very shallow notches the threshold *K*_t_ shifts towards lower values of *K*_t_ and the non-propagating domain starts at very low values of *K*_t_. After increasing the notch value beyond a critical value the non-propagating domain disappears completely. With shallow notches the plastic zone at the root of the notch is much larger with respect to deep notches. The plastic enclave that arises at the notch root of shallow notches would embed the nucleated cracks in this plastic shelter stopping their propagation. With deep notches nucleated cracks fast overcome the small plastic zone and become macro cracks [21]. The effect of the stress gradient is also shown in Figure 15.

The driving forces for crack nucleation and propagation in notched components are respectively the maximum stress at the notch tip and the stress gradient ahead of the notch tip [11]. Low *K*_t_ and high *K*_t_ need the same stress σ* for crack nucleation but different applied loads to have such stress at the notch tip. Furthermore, low *K*_t_ means mild stress gradient and a high applied load needed to reach the nucleation stress σ* at the notch tip; on the other hand high *K*_t_ means steep stress gradient and a low applied load needed to reach the same nucleation stress σ* at the notch tip. Moreover, the steep stress gradient at high *K*_t_ reduces the mean stress at the notch tip (Figure 15). The aggressive environment is another driving force for crack nucleation and, together with the stress gradient and maximum stress, has a big influence on the nucleation process of cracks. The driving force for crack propagation is the applied load: thus the applied stress far from the notch tip (crack tip after nucleation). Such stress is much higher in case of low *K*_t_, thus reducing the fatigue resistance with respect to higher *K*_t_ specimens. An example of the different stress gradients at low and high *K*_t_, having the same maximum stress σ* at the notch tip, is shown in Figure 15.

The results show that the influence of the aggressive environment is more evident at low *K*_t_ because of the higher crack growth rate in NaCl solution and of the higher applied loads, needed to reach the nucleation stress at the notch tip, together with the stress gradient effect (higher mean loads at the tip of the notch as shown in Figure 15).

Static tests are required to complete the research. Such tests would allow to decouple the environmental and stress effects and understand the corrosion fatigue mechanism in terms of chemical and mechanical driving forces.

## 4. Conclusions

Ti-6Al-4V flat smooth and notched specimens were fatigue tested in order to evaluate corrosion fatigue behavior in aqueous environments. Different environmental test conditions, inert, laboratory air and a 3.5 wt% NaCl aqueous solution, were considered and compared in order to quantify the effect of an aggressive environment on the fatigue life of the Titanium alloy. The experimental plan led to the following results:

(1) Tests in lab air and paraffin oil gave approximately the same maximum stresses at nucleation and complete fracture, while a 20% reduction occurred for the tests in 3.5% NaCl solution, for all the values of *K*_t_.
(2) The microstructure consisted in primary alpha and beta-transformed grains due to the performed heat treatment. The tensile results are compatible with such microstructure;
(3) The fatigue limit at 200.000 load cycles was strongly affected by the experimented notches, even if the fatigue life reached a steady state after *K*_t_ = 8–9. This means that if *K*_t_ is higher than 8–9, σ_max_ does not vary for a notched component and the environment does not influence its fatigue resistance. This seems to be a relative important results in case of the aeronautical Ti-6Al-4V components that work in aggressive 3.5% NaCl solution environments.
(4) From the analysis of the fracture surfaces, no significant difference was observed for air or NaCl solution tested samples. This is probably related with the short propagation time required by the fatigue cracks, not enough to make a general corrosion process start.

## Nomenclature

*K*_t_: stress concentration factor
*R*: load ratio
*N*_f_: number of cycles to failure in the final loading block
σ_FL, max_: interpolated fatigue limit (maximum stress over the fatigue cycle) at 2 × 10^5^ load cycles (MPa)
σ_prior, max_: maximum applied stress on minimum cross section in the loading block prior to that of failure (MPa)
σ_final, max_: maximum applied stress on minimum cross section in the failure block (MPa)
σ_limit, max_: limiting maximum fatigue stress at failure on minimum cross section (MPa)
*E*: Modulus of elasticity (MPa)
*T*: Temperature (°C)
*YS*: Yield strength (MPa)
UTS: Ultimate tensile strength (MPa)
